# East Meets West: Tai Chi and Health Promotion

**DOI:** 10.1093/geroni/igaf122.1826

**Published:** 2025-12-31

**Authors:** Timothy Kauffman, Gong Chen, Timothy Kauffman

## Abstract

East Meets West will focus on Tai Chi as an exercise activity that promotes health and wellness. The 1st paper explores the role of Tai Chi in promoting healthy cardiovascular and neuromuscular systems, metabolic processes and delaying aging declines in a university community. This intervention yielded significant improvements in sleep duration and knees osteoarthritis symptoms. The next presentation focuses on Yin-Yang theory in Tai Chi and emphasizes the balance between stability and mobility, energy flow and mental coordination. The 3rd presentation employs qualitative research to study the benefits of Tai Chi in community health. The findings indicate positive effects in lifestyle changes on physical activity, diet, sleep and psychological resilience. The 4th speaker will provide evidence of the benefits of Tai Chi on persons with ankylosing spondylitis. A 12-week intervention showed statistically significant benefits on pain/stiffness (BASDAI), function (BASFI) and spinal mobility. The final speaker investigates the impact of Tai Chi on balance/falls, cognition and motor health in older adults. Emphasis will be given to outcomes from dual task conditions that inform the cognitive-motor interdependence. There will be an opportunity to learn and perform Tai Chi.

